# Economic burden of lung cancer: A retrospective cohort study in South Korea, 2002-2015

**DOI:** 10.1371/journal.pone.0212878

**Published:** 2019-02-22

**Authors:** Soo Min Jeon, Jin-Won Kwon, Sun Ha Choi, Hae-Young Park

**Affiliations:** 1 College of Pharmacy and Research Institute of Pharmaceutical Sciences, Kyungpook National University, Daegu, South Korea; 2 Lung Cancer Center, Kyungpook National University Chilgok Hospital, Daegu, South Korea; CHA University College of Medicine, REPUBLIC OF KOREA

## Abstract

We evaluated the survival rates and medical expenditure in patients with lung cancer using a nationwide claims database in South Korea. A retrospective observational cohort study design was used, and 2,919 lung cancer patients and their matched controls were included. Medical expenditures were analyzed with the Kaplan-Meier sample average method, and patients were categorized into 4 groups by operation and primary treatment method (i.e. Patients with operation: OP = surgery, OP+CTx/RTx = surgery with anti-cancer drugs or radiotherapy; Patients without operation: CTx/RTx = anti-cancer drugs or radiotherapy, Supportive treatment). The 5-year medical expenditure per case was highest in the OP+CTx/RTx group ($36,013), followed by the CTx/RTx ($23,134), OP ($22,686), and supportive treatment group ($3,700). Lung cancer-related anti-cancer drug therapy was the major cost driver, with an average 53% share across all patients. Generalized linear regression revealed that monthly medical expenditure in lung cancer patients, after adjustment for follow-up month, was approximately 3.1–4.3 times higher than that in the control group (cost ratio for OP = 3.116, OP+CTx/RTx = 3.566, CTx/RTx = 4.340, supportive treatment = 4.157). The monthly medical expenditure at end of life was estimated at $2,139 for all decedents, and approximately a quarter of patients had received chemotherapy in the last 3 months. In conclusion, this study presented the quantified treatment costs of lung cancer on various aspects compared with matched controls according to the treatment of choice. In this study, patients with operation incurred lower lifetime treatment costs than patients with CTx/RTx or supportive treatment, indicating that the economic burden of lung cancer was affected by treatment method. Further studies including both cancer stage and treatment modality are needed to confirm these results and to provide more information on the economic burden according to disease severity.

## Introduction

Global lung cancer deaths were estimated at 1.7 million in 2015, contributing to approximately 20% of all cancer-related deaths [1, 2]. As is the case worldwide, lung cancer is the leading cause of cancer deaths in Korea, accounting for 23% of all cancer deaths even though its prevalence share is relatively low at 4.3% of all cancers [3]. This is related to the high mortality rate and delayed diagnosis of lung cancer. In addition, the mortality rate is affected substantially by age, ethnicity, and socioeconomic circumstances [4, 5]. The 5-year net survival rates varied from 2% in Libya to 30% in Japan for patients diagnosed in 2005–2009 [6], and those in Korea were estimated at 16.2% and 25.1% for patients diagnosed in 2001–2005 and 2010–2014, respectively [3].

Recently, expectations have been rising that overall survival rates for lung cancer could increase with the recent emergence of various biological agents for treatment of lung cancer [7, 8]. However, the treatment costs for new biologics are very high if they are prescribed without any restrictions, and their cost-effectiveness should be evaluated to assess the priorities of target patients and to set up the treatment budget [9]. In particular, the economic burden of lung cancer is already very high. In 2004, lung cancer was associated with highest treatment cost at $4.2 billion, making up approximately 20% of the total treatment costs among cancer patients using Medicare in the United States [10], and imposing the greatest burden among all cancers in European countries [11]. Therefore, it is important to analyze current costs for lung cancer and evaluate the areas in which improvement is needed in order to efficiently manage treatment costs in the future.

Currently, various sources are available to analyze the cost of illness, such as claims data, patient records in medical institutes, and survey data [12]. In Korea, most of the population is covered under the national health insurance (NHI) scheme [13]; nationwide sample cohort data sets extracted from the whole claims database are very useful data sources for cost analysis. In a cost study by Shin et al. (2012) [14], they used the NHI claims data for medical expenditure analysis of the major 6 cancers diagnosed in 2006. According to the study, lung cancer patients spent approximately $20,000 for medical costs covered by NHI for 5 years after diagnosis, and the expenditure was highest in regional stage patients [14]. The study broadly covered 6 cancers; however, there is still an unmet need to target lung cancer patients only and to obtain more specific and wider information on the treatment cost for lung cancer. In addition, the outcome of a cost study for lung cancer may also be significantly affected by disease stages, surgical conditions, end-stage care costs, and overall survival [15, 16], and a cost study should consider those aspects. It is also necessary to evaluate whether the cost analysis includes all items or only the diagnostic-specific items, or whether attributable costs or the net difference due to target diseases should be considered in comparison with a control group [12].

Therefore, this study was conducted to assess the following items associated with lung cancer costs in Korea, considering the impact of disease stage by using primary treatment pattern and comparison with a control group; (1) the amount and components of medical expenditure for 5 years after lung cancer diagnosis; (2) the cost ratio between lung cancer patients and matched controls; and (3) the medical expenditure at end of life. This study may help inform cost data needed for public health policy decisions focused on providing cost-effective prevention and treatment of lung cancer.

## Methods

### Database

The data source for this study was the National Health Insurance Service-National Health Screening Cohort (NHIS-HEALS). The NHIS health system covers the entire national population, including social medical insurance (97%) or Medicaid (3%), and provided biennial health screening services for all insured people over 40 years. The NHIS-HEALS database was established by random selection of 10% of patients receiving a medical check-up (40–79 years old) in 2002 and 2003 and followed them until 2015. The database included the results of health screening and data on beneficiary qualifications, including income level, diagnosis, clinical information, and death record. The diagnostic information was recorded using the International Codes of Disease 10th Edition (ICD-10).

### Study design and participant selection

**Fig 1:**
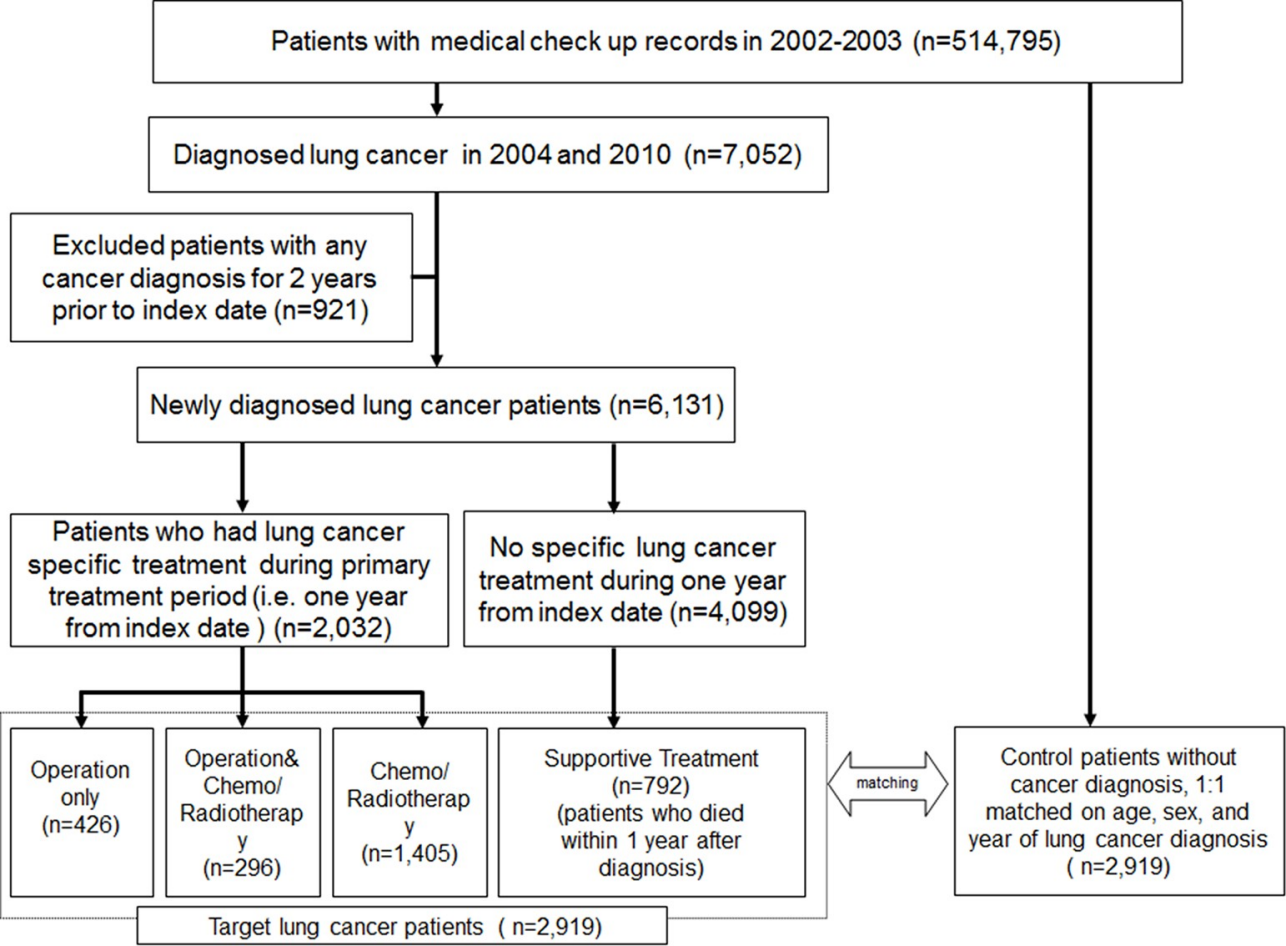
Patient selection scheme.

The study was designed as a retrospective observational cohort study to investigate the survival and medical expenditure according to disease stage and primary treatment pattern in patients with lung cancer in comparison with matched controls. A total of 2,919 lung cancer patients were included for this, and the detailed methods to define the target patients and control group were as follows (Fig 1). The population (n = 7,052) with newly diagnosed lung cancer was classified as patients with ICD-10 codes of C33 and C34 between January 2004 and December 2010 to reserve 5-year follow-up periods. The index date for each patient was defined as the first date of lung cancer diagnosis. To rule out the effect of other cancers, we restricted the study population to those who had no diagnostic record of other cancers for 2 years prior to the index date (n = 921). We defined the first year after diagnosis as the primary treatment period and categorized patient groups by treatment (i.e. OP = surgery; OP+CTx/RTx = surgery with anti-cancer drugs or radiotherapy; CTx/RTx = anti-cancer drugs or radiotherapy). During the primary treatment period, there were 4,099 patients who had no record of lung cancer treatment. Of these patients, if death occurred within 1 year after diagnosis, they were allocated to the supportive treatment group (n = 792); the other patients were considered false-positive cases and were excluded. The selected cases were classified into patients with operation treated by OP or OP+Ctx/Rtx and patients without operation treated by CTx/Rtx or supportive treatment. Control patients were selected from the entire population of the database excluding patients with any kind of cancer, with a 1:1 match for age, sex, and year of lung cancer diagnosis using an exacting matching algorithm. The control was given the same index date as the corresponding lung cancer patient.

### Characteristics of study population

Patient characteristics, such as age, sex, comorbidities, income level, and smoking status, were investigated for 1 year prior to the index date. A Charlson comorbidity index (CCI) indicating comorbidity status was constructed by diagnosis codes from the 1 year before the index date according to a previous paper [17]. Income level was categorized into 5 groups (Medicaid & NHI self-employed/employee subscriber low, NHI self-employed subscriber medium, NHI self-employed subscriber high, NHI employee subscriber medium, and NHI employee subscriber high) based on the insurance amount that the beneficiary qualified for. Information on smoking status was collected using questionnaires at the health status check-up. Smoking status was grouped into ever or current smoker, never-smoker, and missing because of a limitation of the database. The definition of surgery, anticancer therapy (chemotherapy or target therapy) related to lung cancer, and radiotherapy in this study is summarized in supporting information (S1 Table. Definition of Lung cancer specific treatment)

### Medical expenditure for 5 years and end of life

We calculated the total medical expenditure of lung cancer patients according to the primary treatment pattern. Total expenditure was evaluated for 5 years, because all patients were followed for at least 5 years or until death. To reduce the bias associated with early death or loss to follow-up before 5 years, we used the Kaplan-Meier sample average (KMSA) estimator method [18, 19]. This calculation provides a nonparametric estimate of the average expenditures for patients with variable lengths of follow-up evaluation. The KMSA estimator is calculated using the following formula: $Total\;cost(m)=\sum_{t=1}^{m}S(t)C_{t}$ where ‘t’ represents the post-index-date month, S(t) is the survival probability, and Ct is the mean actual cost in period t among patients who survived in month t. In this study, we divided the time interval into months. In addition, the distribution of medical cost by treatment category was analyzed for 1 or 5 years from the index date using the same method used for estimating the total medical expenditure. Major treatment was categorized as the use of pharmaceuticals, surgery, radiotherapy, and other treatments. Pharmaceuticals were categorized as anti-cancer chemotherapy, anti-cancer target therapy, and others.

We defined end of life as the last 3 months prior to the death date. The patients who died during the study period and those who survived more than 3 months after the index date were selected and their medical expenditure was analyzed for the cost of end of life.

In this study, we considered only direct medical expenditure; indirect expenditures (i.e. caregiver expenditures, transportation fee, and productivity loss) were not calculated. Total medical expenditure consists of physician visits, medical procedures, and pharmaceutical expenditures covered by NHI, including insurer reimbursement and patient’s co-payments. Pharmaceutical expenditure includes medication cost and dispensing fees from pharmacists.

### Statistical analysis

The numeric variables were reported as the mean and standard deviation (SD), and frequency and percentage were used for reporting categorical variables. Survival rate was estimated using the Kaplan-Meier method. Estimated expenditure was represented as the mean expenditure adjusted based on the Consumer Price Index for medical care in 2017 and converted into US dollars based on the exchange rate of 2017 (1$ = 1130.48 Korean Won). To assess the impact of lung cancer according to primary treatment method on medical expenditure, generalized linear regression model (GLM) adjusted for age, sex, CCI score, and income level were used. For adjustments of non-normal distribution of medical expenditure, log link and gamma distributions were selected in the GLM. In addition, we reanalyzed an identical GLM after considering the follow-up month as the offset variable to compare the time effect on medical expenditure. SAS enterprise guide 7.1 (SAS Institute Inc., Cary, NC, USA) and R studio version 1.0.136 (R studio, Inc.) was used to perform all analyses.

### Ethics approval and informed consent

The NHIS-HEALS database was retrospectively established in an anonymous format, and the informed consent requirement was waived. The study protocol was approved by the institutional review board of Kyungpook National University (approval number: KNU 2018–0021).

## Results

### Characteristics of the study population

The characteristics of newly diagnosed lung cancer patients are presented in Table 1. The distribution of patients with OP, OP+CTx/RTx, CTx/RTx, and supportive treatment was 14.6%, 10.1%, 48.1%, and 27.1%, respectively. The mean age of all patients was 67.1 (SD: 9.2) years, and 78.1% were male. While the OP+CTx/RTx group was younger in age, the supportive treatment group had a greater number of older people than the other groups (proportion of patients aged 80 or over was 21% vs. 1–4%). Approximately half (51.1%) the patients were ever or current smokers and the average CCI score was 2.3 (SD: 1.3). The CTx/RTx group had the highest proportion of ever or current smokers (54.8%). The distribution of income level was similar in all groups, except for the supportive treatment group. The supportive treatment group had a greater proportion of patients with a low-income level compared to the other treatment groups.

**Table 1:** Characteristics of the study population.

Number of patients (%)	Patients with operation		Patients without operation		Total Cases	Total Controls
	OP	OP+CTx/RTx	CTx/RTx	Supportive treatment		
Total	426(14.6)	296(10.1)	1405(48.1)	792(27.1)	2919(100.0)	2919(100.0)
Males	313(73.5)	221(74.7)	1135(80.8)	611(77.2)	2280(78.1)	2280(78.1)
Age at diagnosis						
Median (interquartile range)	64.5(13.0)	62.0(12.0)	67.0(12.0)	74.0(10.0)	68.0(13.0)	68.0(13.0)
Mean (SD)	63.7(8.9)	62.1(8.3)	65.8(8.6)	73.2(7.7)	67.1(9.2)	67.1(9.2)
40–60 (%)	127(29.8)	114(38.5)	315(22.4)	43(5.4)	599(20.5)	599(20.5)
60–80 (%)	290(68.1)	180(60.8)	1033(73.5)	580(73.2)	2083(71.4)	2083(71.4)
80+ (%)	9(2.1)	2(0.7)	57(4.1)	169(21.3)	237(8.1)	237(8.1)
Year at diagnosis						
2004	58(13.6)	30(10.1)	171(12.2)	132(16.7)	391(13.4)	391(13.4)
2005	46(10.8)	34(11.5)	195(13.9)	105(13.3)	380(13.0)	380(13.0)
2006	44(10.3)	38(12.8)	197(14.0)	105(13.3)	384(13.2)	384(13.2)
2007	59(13.9)	54(18.2)	210(15.0)	109(13.8)	432(14.8)	432(14.8)
2008	75(17.6)	50(16.9)	195(13.9)	124(15.7)	444(15.2)	444(15.2)
2009	72(16.9)	43(14.5)	204(14.5)	98(12.4)	417(14.3)	417(14.3)
2010	72(16.9)	47(15.9)	233(16.6)	119(15.0)	471(16.1)	471(16.1)
Smoking status^**a**^						
Missing	5(1.2)	2(0.7)	22(1.6)	17(2.2)	46(1.6)	33(1.1)
Non-smoker	216(50.7)	148(50.0)	613(43.6)	404(51.0)	1381(47.3)	1801(61.7)
Smoker^**b**^	205(48.1)	146(49.3)	770(54.8)	371(46.8)	1492(51.1)	1085(37.2)
Charlson Comorbidity Index(CCI) score						
Median (interquartile range)	2.0(2.0)	2.0(2.0)	2.0(2.0)	2.0(3.0)	2.0(2.0)	2.0(2.0)
Mean (SD)	2.3(1.3)	2.1(1.2)	2.3(1.3)	2.6(1.4)	2.3(1.3)	2.1(1.3)
1	156(36.6)	122(41.2)	520(37.0)	219(27.7)	1017(34.8)	1264(43.3)
2	116(27.2)	81(27.4)	398(28.3)	227(28.3)	822(28.2)	799(27.4)
3	76(17.8)	55(18.6)	224(15.9)	146(18.4)	501(172)	394(13.5)
4	40(9.4)	20(6.8)	134(9.5)	88(11.1)	282(9.7)	235(8.1)
5	38(8.9)	18(6.1)	129(9.2)	112(14.1)	297(10.2)	227(7.8)
Income level^**c**^						
1	51(12.0)	40(13.5)	233(16.6)	195(24.6)	519(17.8)	523(17.9)
2	64(15.0)	46(15.5)	255(18.2)	163(20.6)	528(18.1)	468(16.0)
3	54(12.7)	44(14.9)	150(10.7)	62(7.8)	310(10.6)	359(12.3)
4	96(22.5)	70(23.7)	360(25.6)	151(19.1)	677(23.2)	653(22.4)
5	161(37.8)	96(32.4)	407(29.0)	221(27.9)	885(30.3)	916(31.4)

OP = surgery; OP+CTx/RTx = surgery with anti-cancer drugs or radiotherapy; CTx/RTx = anti-cancer drugs or radiotherapy

^a^ The most recent health screening results based on the time of diagnosis

^b^ Current smoker or ex-smoker

^c^ 1: Medicaid & National Health Insurance (NHI) self-employed/employee subscriber Low; 2: NHI self-employed subscriber Medium; 3: NHI self-employed subscriber High; 4: NHI employee subscriber Medium; 5: NHI employee subscriber High

### Survival and medical expenditure for 5 years

**Fig 2:**
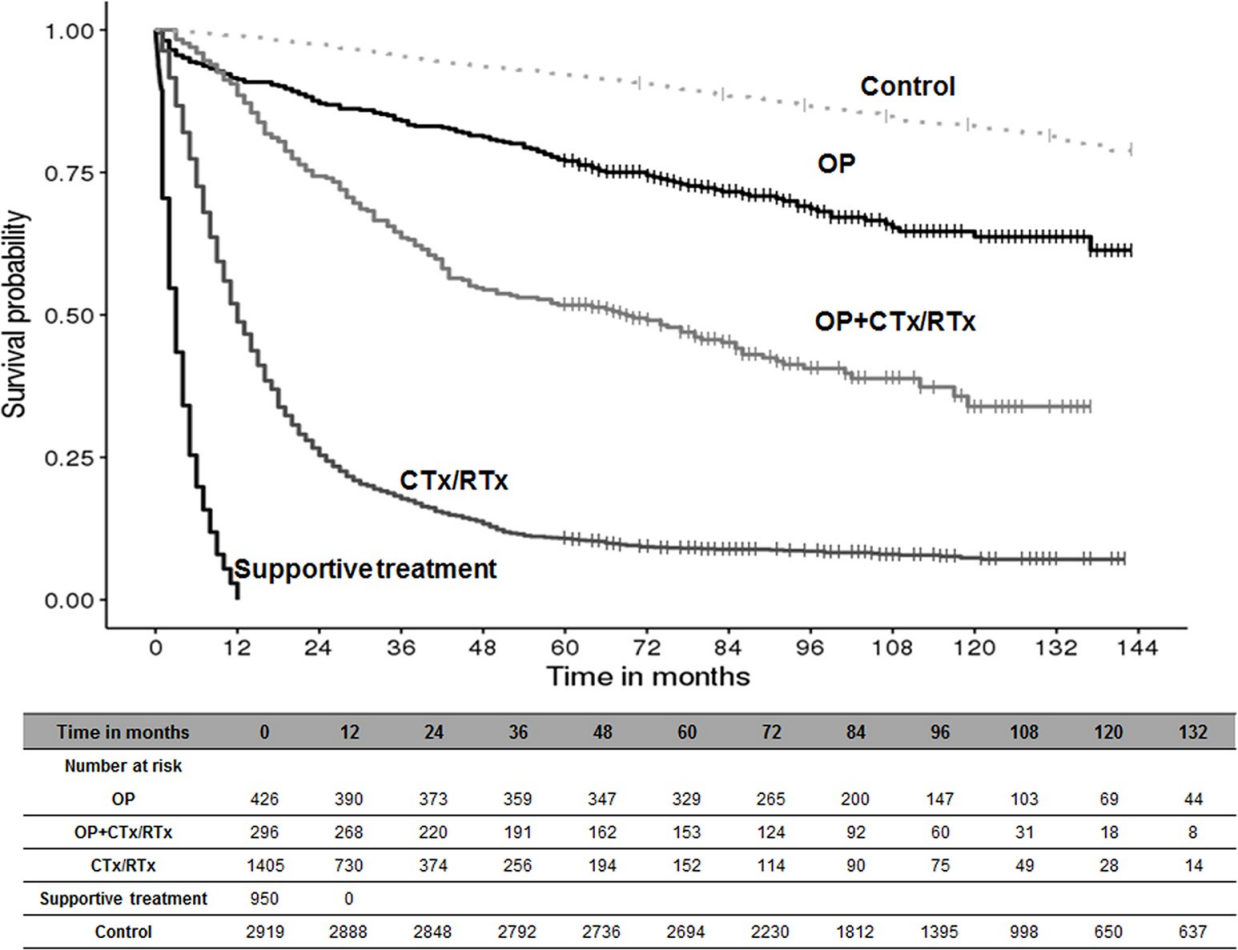
Survival rate by primary treatment pattern. OP = surgery; OP+CTx/RTx = surgery with anti-cancer drugs or radiotherapy; CTx/RTx = anti-cancer drugs or radiotherapy.

The survival rate of patients receiving different types of primary treatment over 10 years is shown in Fig 2. After diagnosis, the 5-year survival rate was 77.0%, 51.7%, and 10.7% in the OP, OP+CTx/RTx, and CTx/RTx groups, respectively. Patients in the OP group had a significantly higher survival rate than those in the other groups, and the median survival time had not yet been reached during the follow-up period. In the supportive treatment group, the survival rate declined sharply and approximately 50% patients died within 3 months. The average total medical expenditure estimate over 5 years for the lung cancer patients was $19,054 per case (Table 2). The 5-year medical expenditure per case in the OP+CTx/RTx group was the highest ($36,013), followed by the CTx/RTx group ($23,134), the OP group ($22,686), and the supportive treatment group ($3,700). Approximately 53% of the 5-year medical expenditure in all patients was associated with lung cancer-related anti-cancer therapy (Table 3). The distribution of medical expenditure was quite different according to primary treatment during the first year. The distribution of anti-cancer therapy in the CTx/RTx group was higher than all other groups in the first year, as expected. The OP group and supportive treatment group did not use anti-cancer therapy in the first year according to the definition; however, if they survived beyond 1 year, they gradually received anti-cancer therapy (15.4% for the OP group over 5 years). Surgery costs in the OP group accounted for approximately 29.6% of costs in the first year and for 13.7% of the 5-year total cost.

**Table 2:** Total medical expenditure for 5 years in lung cancer patients.

		Period	Survival rate (%)	Average cumulative cost ($)
Total cases (n = 2,919)		1 Year	45.8%	11,273
		2 Year	32.4%	14,608
		3 Year	27.2%	16,573
		4 Year	23.8%	18,045
		5 Year	21.7%	19,054
Patients with operation (n = 722)	OP (n = 426)	1 Year	91.3%	10,286
		2 Year	87.1%	13,708
		3 Year	84.0%	16,573
		4 Year	81.2%	19,590
		5 Year	77.0%	22,686
	OP+CTx/RTx (n = 296)	1 Year	88.5%	17,560
		2 Year	74.3%	23,989
		3 Year	63.5%	29,491
		4 Year	54.4%	33,386
		5 Year	51.7%	36,013
Patients without operation (n = 2,197)	CTx/RTx (n = 1,405)	1 Year	48.8%	14,805
		2 Year	25.3%	19,219
		3 Year	17.7%	21,233
		4 Year	13.3%	22,534
		5 Year	10.7%	23,134
	Supportive treatment (n = 792)	1 Year	0.0%	3,700
Total controls (n = 2,919)		1 Year	98.9%	1,374
		2 Year	97.5%	2,955
		3 Year	95.3%	4,731
		4 Year	93.5%	6,564
		5 Year	92.1%	8,438

OP = surgery; OP+CTx/RTx = surgery with anti-cancer drugs or radiotherapy; CTx/RTx = anti-cancer drugs or radiotherapy

**Table 3:** The distribution of medical expenditure by treatment for 1 year or 5 years.

Distribution (%)	Patients with operation				Patients without operation			Total cases	
	OP		OP+CTx/RTx		CTx/RTx		Supportive treatment		
	1 year	5 year	1 year	5 year	1 year	5 year	1 year	1 year	5 year
Pharmaceuticals	20.3%	41.0%	62.0%	64.9%	77.2%	76.1%	30.8%	68.3%	0.0%
*Anti-cancer chemotherapy*	*0*.*0%*	*13*.*2%*	*50*.*8%*	*44*.*4%*	*63*.*0%*	*56*.*0%*	*0*.*0%*	*46*.*4%*	*0*.*0%*
*Anti-cancer target therapy*	*0*.*0%*	*2*.*2%*	*1*.*1%*	*6*.*6%*	*3*.*3%*	*7*.*5%*	*0*.*0%*	*6*.*7%*	*0*.*0%*
*Others*	*20*.*3%*	*25*.*6%*	*10*.*0%*	*13*.*9%*	*10*.*9%*	*12*.*6%*	*30*.*8%*	*15*.*2%*	*0*.*0%*
Surgery	29.6%	13.7%	10.8%	6.5%	0.0%	0.0%	0.0%	2.7%	0.0%
Radiotherapy	0.0%	0.7%	2.3%	1.9%	2.6%	2.2%	0.0%	1.9%	0.0%
Other treatment	50.1%	44.6%	24.9%	26.7%	20.2%	21.6%	69.2%	27.1%	0.0%

OP = surgery; OP+CTx/RTx = surgery with anti-cancer drugs or radiotherapy; CTx/RTx = anti-cancer drugs or radiotherapy

### Impact of lung cancer and primary treatment on medical expenditure

The results of the GLM analysis are presented in Table 4. Apart from the supportive treatment group, lung cancer patients had greater medical expenditure per patient than the control group, and the magnitude of the impact on cost for each treatment group was consistent with the 5-year estimated medical expenditure (cost ratio [CR] for OP = 1.857, CR for OP+CTx/RTx = 2.621, CR for CTx/RTx = 1.464, CR for supportive treatment = 0.266). The monthly medical expenditure in all lung cancer patients based on GLM adjusted for the follow-up month was 3.7 times higher than that in the control group (95%CI: 3.5–4.0). Patients in the CTx/Rtx or supportive treatment group had the highest monthly medical expenditure, was approximately 4.2–4.3 times higher than that of the control group. The OP group had relatively lower monthly medical expenditure per patient than the CTx/RTx group (CR = 3.116 for OP; 95% CI 2.842–3.417 vs. CR = 4.340 for CTx/RTx; 95% CI 3.990–4.720).

**Table 4:** Results of generalized linear regression analysis on medical expenditure.

Variable		Total medical expenditure per study period^a^			Medical expenditure per month^b^		
		Cost ratio	95% LCL	95% UCL	Cost ratio	95% LCL	95% UCL
Control (Reference)		1.000			1.000		
Lung cancer (all)		1.395	1.312	1.483	3.739	3.483	4.012
Control (Reference)		1.000			1.000		
Patients with operation	OP	1.857	1.704	2.025	3.116	2.842	3.417
	OP+CTx/RTx	2.621	2.359	2.914	3.566	3.193	3.983
Patients without operation	CTx/RTx	1.464	1.364	1.572	4.340	3.990	4.720
	Supportive treatment	0.266	0.245	0.289	4.157	3.786	4.564

LCL: lower confidence limits; UCL: upper confidence limits; OP = surgery; OP+CTx/RTx = surgery with anti-cancer drugs or radiotherapy; CTx/RTx = anti-cancer drugs or radiotherapy

^**a**^ Effects are shown as adjusted for sex, age, smoking status, CCI score, and income level

^**b**^ Effects are shown as adjusted for sex, age, smoking status, CCI score, income level, and length of follow-up month

### Medical expenditure at end of life

**Fig 3:**
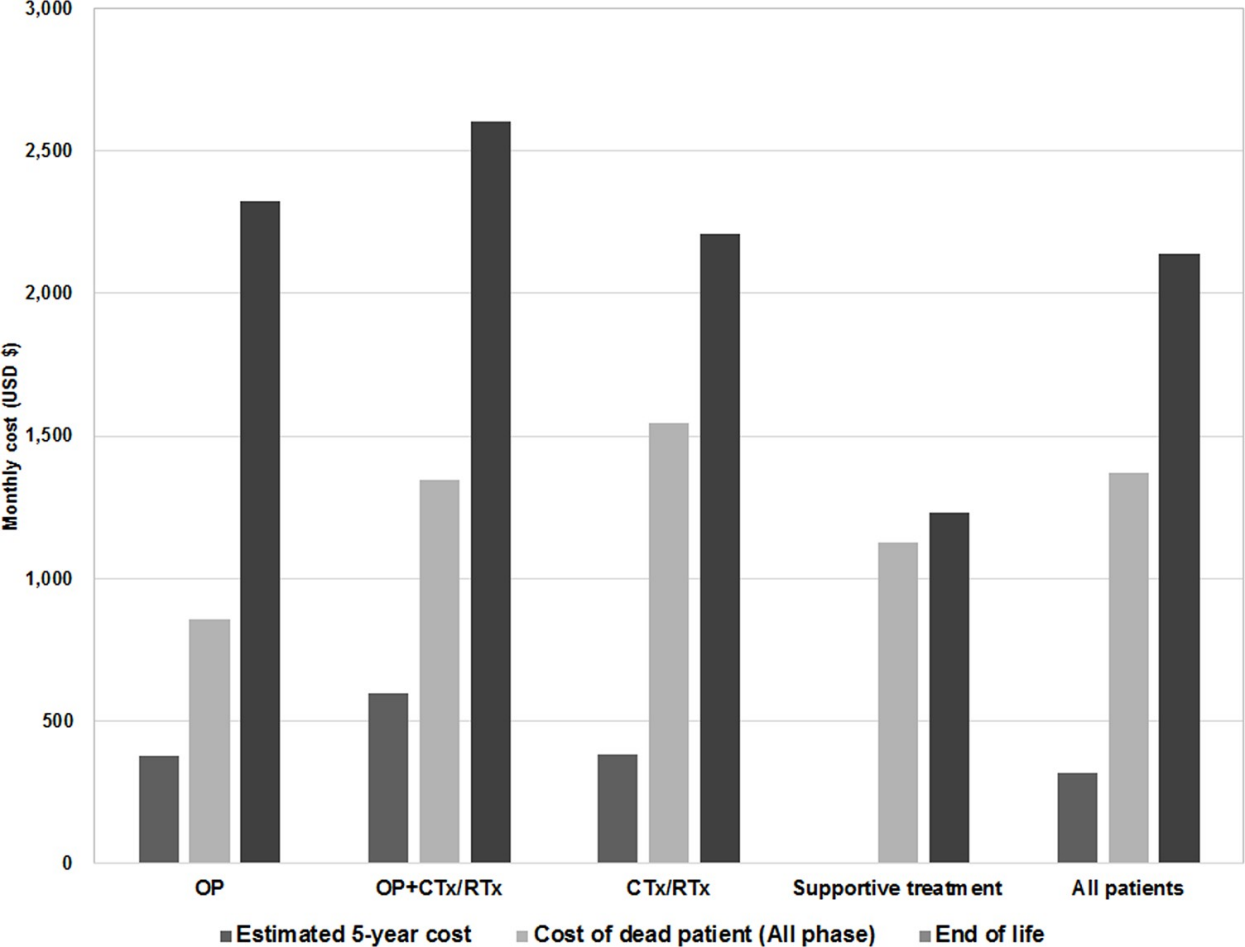
Monthly medical expenditure for end of life by treatment pattern. OP = surgery; OP+CTx/RTx = surgery with anti-cancer drugs or radiotherapy; CTx/RTx = anti-cancer drugs or radiotherapy.

**Fig 4:**
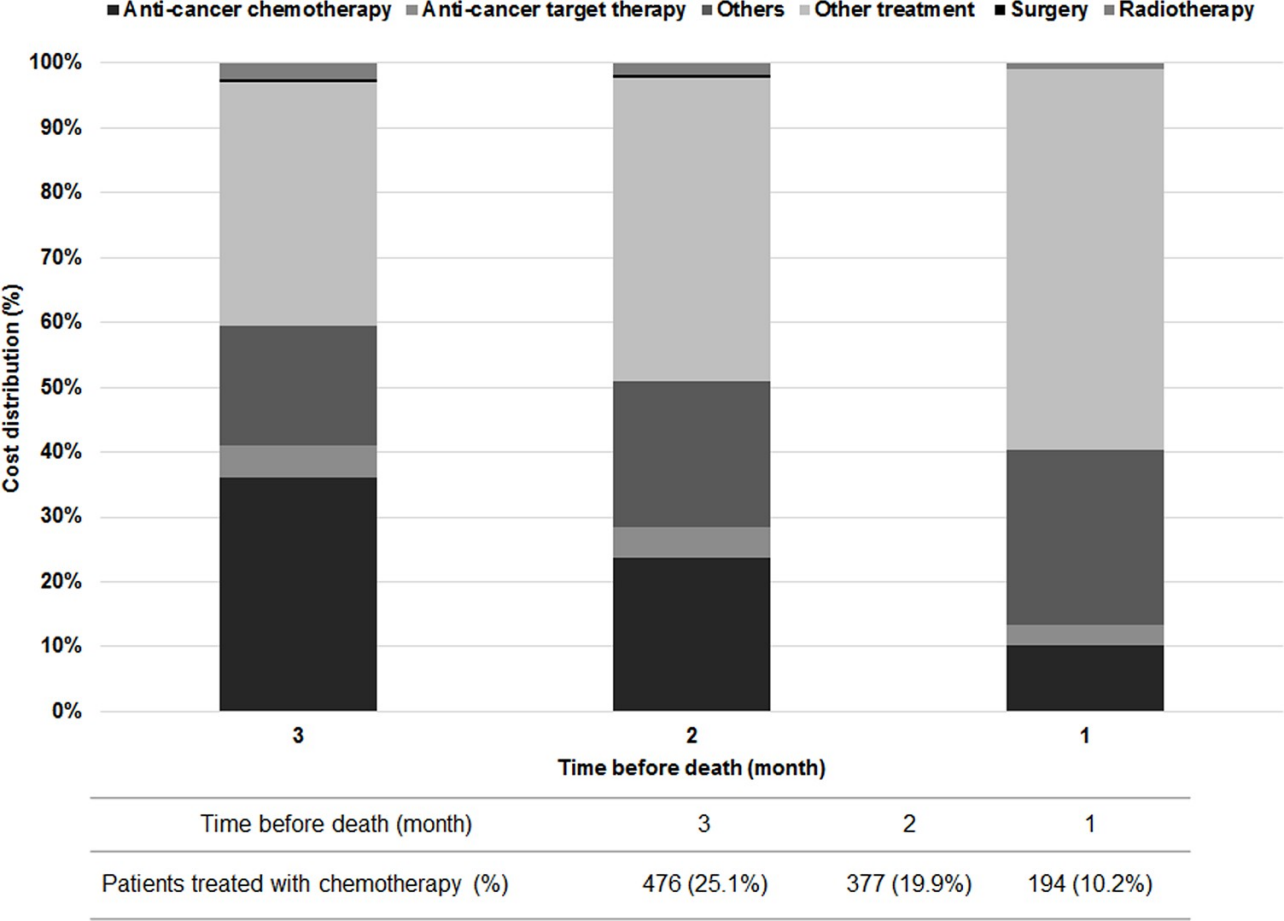
Distribution of medical expenditure by treatment method for end of life.

The monthly medical expenditure at end of life was $2,139 for all decedents, and it tended to be much higher than the monthly medical expenditure for overall 5-year cost in all phase patients ($318) (Fig 3). Although the proportion of anti-cancer treatment related to lung cancer in medical expenditure decreased near time of death, it was the greatest cost driver in the end stage. While the proportion of other treatments was gradually increased, that of anti-cancer treatment tended to decrease because the number of anti-cancer therapy users also decreased. The number of people receiving anti-cancer treatment related to lung cancer was 476 patients (25.1%), 377 patients (19.9%), and 194 patients (10.2%) in the last 3 months (Fig 4).

## Discussion

In this study, we found that lung cancer patients had approximately 4 times higher monthly medical expenditure compared to matched controls during their lifetime. The shorter the life span, the higher the monthly average cost, and it was confirmed that operation significantly affect medical expenditure, as well as survival. Additionally, various cost details for lung cancer were obtained, such as the proportion of drug costs, expenditure differences by treatment pattern and phase, and increased costs and proportions of chemotherapy at the end of life.

The KMSA method was used to analyze costs in this study because the estimates closest to the actual values could be analyzed by including censored data with this method [20]. The total cumulative medical expenditure for 1 year and 5 years was estimated to be approximately $10,000 and $20,000 per person, respectively. The expenditure for 1 year after diagnosis corresponds to approximately 30% of South Korea's GDP per capita of $29,891 in 2017. The absolute costs, as calculated in this study, appear to be lower than those described in studies conducted in other countries. A study analyzing the costs of 1,210 non-small cell lung cancer (NSCLC) patients using claims data from a private health insurance company in the USA showed that the average monthly cost after a diagnosis of lung cancer was $16,577 (SD: 33,350). Approximately 85% of the medical expenditure incurred over 5 years in Korea was spent in 1 month in the USA [16]. In a study comparing the costs of lung cancer among 3 European countries, higher costs compared to Korea were also identified, as the average treatment costs for 2 years were €17,777, €25,063, and €32,500 for the United Kingdom, France, and Germany, respectively [21].

However, each study on the cost of illness has different demographic characteristics, cost scope, and observation period depending on the analysis method and the database used, making it difficult to compare results directly. In addition, the absolute value of lung cancer treatment cost can be greatly influenced by the socioeconomic environment, such as the nation's medical settings, income level, and government subsidization. Thus, we conducted a cost comparison between lung cancer patients and matched controls using GLM analysis and determined the cost ratio between groups. The average monthly costs and total costs were compared as the total cost varies considerably depending on the survival time, and the monthly costs tend to be higher in patients with shorter life spans according to the GLM analysis.

In this study, patients were divided into 4 groups by using primary treatment pattern based on the National Comprehensive Cancer Network guidelines [22]. Patients in the OP group, who can be described as having early stage disease, had a relatively higher expenditure in the initial phase mainly due to surgery; however, the monthly average costs in this group were the lowest among the 4 subgroups, mainly owing to the longer survival period. However, this result may not be applicable if the reimbursement conditions are very different to those in South Korea or the medical cost of surgery is very high. The OP+CTx/RTx group, which is estimated to contain patients with stage 1B–3A, showed the highest expenditure. This group was assumed to have received adjuvant or neo-adjuvant therapy in addition to surgery, and anti-chemotherapy treatments could have increased the total medical expenditure. The CTx/RTx group had the highest cost per month of survival, costing approximately 1.4 times more than the lowest cost group of OP. In a study by Schwarzkopf et al. (2015), the average cost per year survived for the CTx/RTx group was approximately 1.7 times higher than other treatment groups, indicating a similar trend to this study [23]. Since survival is a good endpoint for the effectiveness of cancer treatments, medical costs per month survived could be an indicator of cost-effectiveness. Early diagnosis and surgery of lung cancer has a significant impact on increased survival [24, 25], and the results of this study indirectly demonstrate that early diagnosis is a cost-effective strategy for the management of lung cancer. Our study showed that 27.1% of all participants were not actively treated for lung cancer. Other studies also included groups of patients who received supportive care only, such as 20.5% of patients in a German study involving 17,478 patients with lung cancer[23] and 27.7% of squamous NSCLC patients in a USA study [26]. The median survival of these patients was only a few months; 3 months (Korea), 0.9 months (Germany), and 2 months (USA). It is thought that most patients lost treatment time because of the delayed diagnosis of lung cancer [27], and the treatment had to be stopped because of old age in patients aged 80 years or more (approximately 17% of this study). The relatively high proportion of patients in the supportive treatment group also supports the need for early diagnosis not only to increase the life span but also to achieve cost-effective management of lung cancer.

Drug costs were the highest expenditure component in all subgroups except for the expenditure in the first year of the OP group. These results were consistent with the those of a previous study by Shin et al. (2016) in which 72.6% of total costs were used for drugs [14]. Although there was no expenditure for drug therapy in the first year after diagnosis in the OP group, the drug costs increased to 13.2% in the total 5-year expenditures. This is most likely associated with chemotherapy, which might be initiated in the event of recurrences after surgery. Frequent recurrences increase the cost of chemotherapy and threaten survival, and therefore, a major aim is to prevent recurrences after surgery [28, 29]. The exact proportion of costs resulting from recurrences could not be analyzed in this study, and a detailed analysis of recurrence-related costs should be considered for future studies. Meanwhile, gefitinib was introduced in Korea in 2004, but its usage was limited to second line treatment until 2010; thus, the proportion of patients receiving targeted therapeutic agents might be small in our analysis. Reimbursement coverage was extended for first line therapy in patients with epidermal growth factor receptor mutation from 2011, and new biologic agents were introduced more recently. Therefore, it is expected that there will be a further increase in the cost of drug therapies from 2011. Studies using real-world data considering their costs and effects on survival will be needed to ensure that these expensive agents are affordable and cost-effective compared with conventional agents.

The cost of end of life for patients with cancer is reported to be higher than that of patients without cancer [30]. In this study, the medical expenditure at end of life was approximately 2 times or 7 times higher than the monthly average of the whole observation period for decedents or all patients, respectively. The costs were higher in the CTx/RTx and supportive treatment groups. This might be related to the short survival period and chemotherapies that were implemented until the end of life. This study found that 25.1% of decedents received chemotherapy during the last 3 months of life, and 10.2% of patients received chemotherapy in the last month before their death (Fig 3). The guidelines by the American Society of Clinical Oncology do not recommend chemotherapy for patients with end stage disease and poor performance status [31]. Furthermore, a recent study reported that even patients with a good performance status had poorer quality of life than those who did not receive chemotherapy (odds ratio: 0.34, 95% CI: 0.17, 0.75) [32]. Thus, the use of chemo-agents should be carefully considered as the quality of life and palliative care of the patient must be taken into account at the end of life [33, 34].

This study has several limitations. First, the details on disease stage (i.e. stage I, stage II, stage III, or stage IV) which is the most important prognostic factor for survival were not included owing to lack of information. Instead, patients were categorized by treatment pattern which was determined by several factors including disease stage. Therefore, the study results should be interpreted with caution, because the economic burden of lung cancer differs according to the stage of lung cancer rather than treatment pattern. Second, lung cancer was defined based on ICD-10 codes in the database and there were potential uncertainties in the diagnosis. In addition, NSCLC and SCLC, which have quite different costs and survival rates, could not be separated. Approximately 80–85% of lung cancers in Koran patients are NSCLC [35]; therefore, the results of this study may be more generalizable to NSCLC. Third, the data source for this study only included patients aged 40 years or older with medical records. The age of the target patients may not affect the analysis results significantly as the proportion of lung cancer patients under 40 years is less than 1.5% [36]; however, the medical check-up history could have a small impact on survival outcomes [37]. Nevertheless, we do not believe that selection bias distorted the overall results, and the results of GLM in comparison with controls could compensate for this bias. Forth, cost items might be allocated incorrectly because there was the possibility of misclassification when they were claimed from medical institutes. Lastly, the results of this study did not include patients’ out-of-pocket costs, which were not covered by government insurance and non-medical costs, such as patient care or transportation costs. In Korea, the out-of-pocket costs were assumed to be 15–20% of the total insurance cost for cancer patients [38]. Based on the research by Park et al., lung cancer showed the highest share of out-of-pocket costs among cancers, and direct non-medical costs accounted for approximately 30% of total direct medical expenditure for caregivers, transportation, and complementary and alternative medicine [39]. Considering all these costs, the actual total cost of lung cancer is estimated to be 1.5 times (approximately $30,000) higher than that estimated in this study.

Many studies have been published on the cost of lung cancer; however, few studies compared results between lung cancer patients and matched controls. Our research investigated both survival and cost outcomes and increased costs of lung cancer compared with controls. We aimed to increase the comparability and utility of study results by presenting the cost ratio for both total costs and the average cost per month of survival between target patients and controls. Therefore, these results are applicable not only for Korea but also for other countries.

In conclusion, this study presented various cost details for lung cancer, and confirmed that the economic burden of lung cancer patients was significantly higher than that of matched controls; in addition, it was affected by treatment methods, which was dependent on multiple factors such as stage of the cancer, and overall health. Patients with operation who might be estimated to be at an earlier stage, survived longer, and their lifetime medical expenditures were lower than patients with CTx/RTx or supportive treatment. However, further studies using clinical information would be required to determine the economic burden of lung cancer according to disease stage. In particular, lung cancer screening is to be implemented urgently for high-risk patients considering the high proportion of untreated patients in this study. The societal and economic burden of lung cancer is very high, and therefore, an in-depth study on mortality and costs could support the identification of unmet public health needs and contribute to relieving socioeconomic burdens from cancer.

## Supporting information

S1 Table Definition of lung cancer specific treatment. (DOCX)
