# Supplementary material for: Economic burden of lung cancer: A retrospective cohort study in South Korea, 2002-2015
Source: PLoS One. 2019 Feb 22;14(2):e0212878. doi: 10.1371/journal.pone.0212878 (PMC6386401; doi:10.1371/journal.pone.0212878)
Supplement: S1 Table — (DOCX) [file pone.0212878.s001.docx]

**S1. Definition of Lung cancer specific treatment**

| Treatment | Treatment Definition |
| --- | --- |
| Surgery | Segmental Resection(O1311-O1316, O1341-O1345, O1410),  Wedge resection(O1401, - O1403), Lobectomy(O1421-O1424),  Pneumonectomy(O1431-O1432), Cryosurgical Ablation(O1471), Mediasternal Lymph Node Dissection(O1596) |
| Chemotherapy | Carboplatin(123701BIJ - 123708BIJ, 123730BIJ-123735BIJ), Cisplatin(134501BIJ - 134503BIJ), Cyclophosphamide(139001ATB),  Docetaxel(148301BIJ-148306BIJ), Doxorubicin(149401BIJ-149405BIJ, 149430BIJ-149435BIJ), Etoposide(157101BIJ, 157103BIJ-157108BIJ),  Gemcitabine(164901BIJ- 164903BIJ), Ifosafimide(173301BIJ), Irinotecan(177401BIJ-177407BIJ, 177430BIJ, 177431BIJ-177437BIJ), Mitomycin(196401BIJ- 196403BIJ), Paclitaxel(207801BIJ- 207806BIJ, 207830BIJ- 207836BIJ), Vinblastin(247801BIJ, 247830BIJ) Vincristine(248001BIJ, 248003BIJ, 248030BIJ), Vinorelbine(248031BIJ, 248201BIJ, 248202BIJ), Pemetrexed(481201BIJ, 481202BIJ) |
| Target therapy | Erlotinib(453001ATB), Gefitinib(477401ATB- 477403ATB) |
| Radiotherapy | Teletherapy(HD051- HD056), Rotational Irradiation(HD057- HD059), 3-Dimennsional Conformal Therapy(HD061),Unsealed Source(HD071-HD073),  Brachytherapy(HD080- HD089), Total Body Irradiation(HD091-HD093),  Fractionated Stereotactic Radiosurgery(HD110- HD112), Cranial Stereotactic Radiosurgery(HD113-HD115), Proton Therapy(HD121), Body Stereotactic Radiosurgery(HD211, HD212), Intensity Modulated Radiation Therapy(HZ271) |
